# Oncological Outcomes of Open Radical Retropubic Prostatectomy in Ireland: A Single Surgeon's 5-Year Experience

**DOI:** 10.1055/s-0038-1675827

**Published:** 2018-11-28

**Authors:** Stefanie M. Croghan, Deep Mudit Matanhelia, Ann T. Foran, David J. Galvin

**Affiliations:** 1 Department of Urology, The Mater Misericordiae University Hospital, Eccles Street, Dublin, Ireland; 2 Department of Urology, St. Vincent's University Hospital, Elm Park, Dublin, Ireland; 3 Department of Urology, St. Vincent's Private Hospital, Elm Park, Dublin, Ireland

**Keywords:** radical retropubic prostatectomy, RRP, open prostatectomy, prostate cancer, oncological outcomes

## Abstract

**Objectives** There is a little published data on the outcomes of radical prostatectomy in the Irish context. We aimed to determine the 5-year oncological results of open radical retropubic prostatectomy (RRP) performed by a single surgeon following appointment.

**Methods** A retrospective review of RRPs performed between 2011 and 2016 was conducted. Patient demographics, preoperative parameters (clinical stage on digital rectal exam, prostate-specific antigen (PSA) levels, biopsy Gleason's score and MRI [magnetic resonance imaging] findings), pathological variables (T-stage, Gleason's score, nodal status, and surgical margin status), and treatment decisions (lymphadenectomy or adjuvant radiotherapy) were recorded. Oncological outcome at last follow-up was ascertained.

**Results** 265 patients underwent RRP between 2011 and 2016. Median age was 62 years (range: 41–74). Mean follow-up was 32.24 months (range: 8–72) months. Pathological disease stage was T2 in 170/265 (64.15%), T3a in 65/265 (24.53%), and T3b in 30/265 (11.32%). Final Gleason's score was upgraded from diagnostic biopsy in 16.35% (43/263) and downgraded in 27% (71/263). Pelvic lymph node dissection was performed in 44.25% (118/265) patients. A positive surgical margin (PSM) was seen in 26/170 (15.2%) patients with T2 disease and in 45/95 (47.37%) patients with T3 disease. Of the 265 patients, 238 (89.81%) were disease-free at last follow-up, of whom 24/238 (10.08%) had received adjuvant and 17/238 (7.14%) received salvage radiotherapy. Adjuvant/salvage treatment was ongoing in 19/265 (7.17%) of patients.

**Conclusion** Good oncological outcomes of RRP in the Irish context are seen in this 5-year review, with the vast majority of patients experiencing biochemical-free survival at most recent follow-up.

Prostate cancer is reported to be the most common invasive cancer affecting both Irish and European men overall (excluding non-melanoma skin cancers). 1 2 Over 3,400 Irish men are diagnosed per annum, with National Cancer Registry Ireland (NCRI) figures estimating that 28% of men proceed to surgical treatment within a year of diagnosis (2012–2014). 3 This figure has been steadily increasing and may in part reflect a trend toward early-stage diagnosis facilitated by Rapid Access Prostate Cancer Clinics (RAPCs) 4 which were established in eight national cancer centers following the Irish National Cancer Control Program (NCCP), National Strategy for Cancer Control 2006. 5

While radical prostate surgery has evolved greatly over the past decade, with a variety of techniques now established in urological practice, open radical prostatectomy remains a common procedure. Surprisingly, however, there is a paucity of data regarding Irish outcomes of this approach.

The aim of the present study was to evaluate the oncological outcomes of patients undergoing open radical retropubic prostatectomy (RRP) performed by a single surgeon across three centers in Ireland.

## Methods

All patients who underwent open RRP, with or without pelvic lymph node dissection (PLND), performed by a single surgeon (D.J.G.) between September 2011 and November 2016 were included. Surgery was performed on one of three sites–The Mater Misericordiae University Hospital, St. Vincent's University Hospital, and St. Vincent's Private Hospital, all located in Dublin, Ireland.

Our practice is to offer surgical treatment to all patients with non-metastatic prostate cancer considered fit for surgery, having counseled them as to alternative options, including radiotherapy (with external beam radiotherapy or brachytherapy discussed by a radiation oncologist) and, where appropriate, active surveillance (AS). The absence of metastatic disease is confirmed on clinical assessment and radiological staging with multiparametric MRI prostate with additional isotope bone scan +/− CT (computed tomography) TAP (thorax abdomen pelvis) in patients with Gleason score > 7 on biopsy or prostate-specific antigen (PSA) ≥10 mg.

The decision to perform pelvic lymph node dissection (PLND) is based on preoperative predicted rates of lymph node positivity, for which we routinely use Memorial Sloan Kettering Cancer Centre (MSKCC) nomograms, 6 proposing PLND to patients in categories with ≥5% estimated rates of nodal positivity. The form of lymphadenectomy performed during the study period was standard PLND, comprising obturator and external iliac nodal basins with extended nodal dissection (ePLND) performed on selected patients (generally prompted by young age or significant burden of high grade disease). A nerve-sparing approach is attempted where clinical staging (taking into account preoperative imaging and intraoperative findings) is estimated at ≤T2; patients with ≥T3 diseases are considered for a unilateral nerve-sparing approach on the contralateral side in cases where extra-capsular extension is appreciated on one side of the gland only.

All patients are discussed postoperatively at a urology multidisciplinary team (MDT) meeting, with decision for further treatment, primarily adjuvant radiotherapy, made in this forum. Standard follow-up comprises clinical review with a PSA reading at 3-months and then 6 monthly for 2 years, assuming biochemical recurrence is not detected. Annual PSA monitoring is adopted thereafter.

A retrospective electronic chart review of all patients was performed. Preoperatively recorded data included: patient age at diagnosis, clinical stage on digital rectal exam, preoperative prostate-specific antigen (PSA) level, Gleason's score and number of positive cores at transrectal ultrasound guided (TRUS) biopsy, and modality and results of imaging performed for radiological staging. The date of surgery and decision regarding the need to perform PLND was documented. Final histopathological results were reviewed with pathological T-stage, Gleason's score, nodal status, and surgical margin status recorded. Surgical margin status was defined as the presence of tumor at the inked surface of the specimen 7 and classified as focal (≤3 mm) or extensive (≥3 mm or multifocal) based on stratification proposed by other authors. 8 9 All patients' oncological outcome was ascertained by review of PSA at last follow-up, and/or imaging findings were relevant. Where a PSA value within the previous 12 months was not available from the chart, an attempt to obtain more recent follow-up data was made by telephoning the patient's general practitioner. Biochemical recurrence was defined as two rising PSA readings ≥0.05. Postoperative radiotherapy was defined as adjuvant if the decision to administer radiotherapy was made at the postoperative MDT meeting (in which case treatment was typically commenced at 4 to 6 months postoperatively) and as salvage if the patient was referred for radiotherapy in response to rising PSA levels where the MDT decision had been for surveillance in the first instance.

## Results

A total of 265 patients underwent open RRP in the study period between 2011 and 2016. Mean age at diagnosis was 61.26 years (range: 41–74 years). Disease characteristics (Gleason's score on TRUS biopsy and PSA at diagnosis) are shown in Table 1 . The majority of our patients (182/265, 68.68%) had Gleason's score 7 disease. Overall, the mean PSA at diagnosis was 9.46 (range: 1.06–76.7). Follow-up is outlined in Table 2 .

**Table 1:** Age and preoperative disease characteristics

Median age	62 y (range: 41–74 y)	
Gleason's (TRUS biopsy)	n (%)	Mean PSA at diagnosis
6	28 (10.56)	8.82 (3.7–35.2)
7	139 (52.45)	8.62 (1.06–29)
8	72 (27.38)	10.22 (2.7–76.7)
9	23 (8.68)	13.19 (3–41)
10	3 (1.13)	6.89 (6.5–7.15)

Abbreviations: PSA, prostate-specific antigen; TRUS, trans-rectal ultrasound.

**Table 2:** Follow-up timeframe

Follow-up	
Mean overall follow-up	32.24 months (8–72)
Mean follow-up post salvage radiotherapy	25.95 months (1–55)

Distribution of Gleason's score, including numbers upstaged or downstaged from TRUS biopsy diagnosis in final histology and T-stage across our patient population is outlined in Table 3 .

**Table 3:** Final histopathology

Histopathology		*n* = 265 (%)
**Final Gleason's score**	6	23 (8.68)
	7	182 (68.68)
	8	29 (10.94)
	9	30 (11.32)
	10	1 (0.38)
	Gleason's upstaged	43/263 (16.35)
	Gleason's downstaged	71/263 (27)
**Pathological T-stage**	T2a	22 (8.30)
	T2b	9 (3.4)
	T2c	139 (52.45)
	T3a	65 (24.53)
	T3b	30 (11.32)

A pelvic lymph node dissection was performed in 118/265 (44.25%) with mean number of nodes retrieved 6.08 (range: 1–22). Lymphadenectomy data are outlined in Table 4 . Of 104 patients staged pN0 (pathologically node negative), 4 had had radiological suspicion of nodal involvement preoperatively. Of these four patients, three received no additional therapy and one received adjuvant radiotherapy; all four were disease-free at last follow-up.

**Table 4:** Lymphadenectomy data

Abbreviations: PLND, pelvic lymph node dissection; LN, lymph node.

The overall rate of positive surgical margins (PSMs) was 26.79% (71/265). As related to T-stage, PSMs were observed in 26/170 (15.2%) of T2 disease (focal in 19/26 [73.07%]) and in 45/95 (47.37%) of T3 disease (focal in 27/45 [60%]). Table 5 describes details of surgical margin status.

**Table 5:** Breakdown of surgical margin status

**T2 (** ***n*** ** = 170)**		
Positive surgical margin: 15.2% (26/170)		
pT stage	Margin status by stage	
T2a ( *n* = 22)	R0	90.9% (20/22)
	R1 (focal)	9.09% (2/22)
	R1 (extensive)	–
T2b ( *n* = 9)	R0	88.89% (8/9)
	R1 (focal)	–
	R1 (extensive)	11.12% (1/9)
T2c ( *n* = 139)	R0	83.45% (116/139)
	R1 (focal)	12.23% (17/139)
	R1 (extensive)	4.32% (6/139)
**T3 (** ***n*** ** = 95)**		
Positive Surgical Margin: 47.37% (45/95)		
pT Stage	Margin status by stage	
T3a ( *n* = 65)	R0	52.3% (34/65)
	R1 (focal)	27.7% (18/65)
	R1 (extensive)	20% (13/65)
T3b ( *n* = 30)	R0	53.34% (16/30)
	R1 (focal)	30% (9/30)
	R1 (extensive)	16.67% (5/30)

At most recent follow-up, 238/265 (89.81%) of patients were disease-free of whom 24/238 (10.08%) had received adjuvant and 17/238 (7.14%) received salvage radiotherapy, and no patients had died of prostate cancer. Of the overall cohort, 33/265 (12.45%) of patients received adjuvant therapy. Of patients that did not receive adjuvant therapy up front ( *n* = 232), 32 patients (13.79%) were ultimately referred for salvage therapy. Either adjuvant or salvage treatment (radiotherapy or androgen deprivation therapy) was ongoing in 19/265 (7.17%) of patients. Mean overall follow-up was 32.24 months (range: 8–72 months).

Overall oncological outcomes relative to preoperative PSA, pathological T and N-stage, final Gleason's score and surgical margin status are outlined in Tables 6 , 7 , 8 .

**Table 6:** Outcomes of pT2 disease

pT stage	Gleason's score	Treatment		Mean PSA	PLND	N1 status (of PLND)	PSM (R1)	Disease-free	Alive with disease	Treatment ongoing*	Others
T2 ( *n* = 170)	6 ( *n* = 22)	Surgery alone	22/22	–	–	–	–	21/22	0/22	–	1/22 died unrelated
		+ Adjuvant	–	–	–	–	–	–	–	–	–
		+ Salvage	–	–	–	–	–	–	–	–	–
	7 ( *n* = 129)	Surgery alone	116/129	8.22 (1.06–29)	29/116	0/29	14/116	114/116	–	–	1/116 lost to follow-up 1/116 died unrelated
		+ Adjuvant	4/129	7.77 (4.6–12.8)	2/4	0/2	3/4	4/4	–	–	–
		+ Salvage	9/129	9.7 (5.8–15.1)	2/9	0/2	4/9	6/9	–	3/6	–
		> Mean time to BCR (mo): 14 (3–39)									
	8 ( *n* = 11)	Surgery alone	8/11	8.28 (2.7–15)	7/8	0/7	3/8	8/8	–	–	–
–		+ Adjuvant	−	–	–	–	–	–	–	–	–
		+ Salvage	3/11	5.6 (4–8.6)	2/3	0/2	0/3	3/3	–	–	–
		> Mean time to BCR (mo): 20 (7–27)									
	9 ( *n* = 7)	Surgery alone	4/7	11.38 (4.3–26.3)	3/4	0/3	0/4	4/4	–	–	–
		+ Adjuvant	1/7	7	1/1	0/1	1/1	1/1	–	–	–
		+ Salvage	2/7	10.4 (10–10.8)	1/2	1/1	0/2	1/2	–	1/2	–
		> Mean time to BCR (mo): 23.5 (5–42)									
	10 ( *n* = 1)	+ Adjuvant	1/1	6.3	1/1	1/1	0/1	1/1	−	−	−

Abbreviations: BCR, biochemical recurrence; PSA, prostate-specific antigen; PLND, pelvic lymph node dissection; PSM, positive surgical margin.

**Table 7:** Outcomes of pT3a disease

pT stage	Gleason's score	Treatment		Mean PSA	PLND	N1 status (of PLND)	PSM (R1)	Disease-free	Alive with disease	Treatment ongoing a
T3a ( *n* = 65)	6 ( *n* = 1)	Surgery alone	1/1	5	0/1	−	0/1	1/1	–	–
–		+ Adjuvant	–	−	–	–	–	–	–	–
		+ Salvage	–	–	–	–	–	–	–	–
	7 ( *n* = 40)	Surgery alone	31/40	8.03 (3–18.12)	13/40	0/13	11/31	31/31	–	–
		+ Adjuvant	2/40	8.1 (6.1–10.1)	2/2	0/2	2/2	2/2	–	–
		+ Salvage^	7/40	9.98 (4.1–16.8)	5/7	0/5	5/7	3/7	–	4/7
		^Mean time to BCR (mo): 20 (5–40)								
	8 ( *n* = 10)	Surgery alone	4/10	7.3825 (6.4–9.48)	4/4	0/4	1/4	4/4	–	–
		+ Adjuvant	4/10	20.42 (8–35.2)	3/4	2/3	4/4	2/4	–	2/4
		+ Salvage^	2/10	16.3 (7.8–24.8)	1/2	0/1	1/2	1/2	–	1/2
		^Mean time to BCR (mo): 18.5 (9–28)								
	9 ( *n* = 14)	Surgery Alone	6/14	10.45 (5.8–23.6)	5/6	0/5	2/6	6/6	–	–
		+ Adjuvant	3/14	29.7 (5.1– 76.7)	3/3	1/3	2/3	3/3	–	–
		+ Salvage^	5/14	19.64 (5.6–41)	4/5	0/4	3/5	2/5	1/5	2/5
		^Mean time to BCR (mo): 26 (6–48)								

a Radiation therapy ongoing, or no evidence disease post radiotherapy but completing prolonged course of androgen deprivation therapy.

**Table 8:** Outcomes of pT3b disease

pT stage	Gleason's score	Treatment		Mean PSA	PLND	N1 status (of PLND)	PSM (R1)	Disease-free	Alive with disease	Treatment ongoing a
T3b ( *n* = 30)	7 ( *n* = 13)	Surgery alone	4/13	13.03 (5.6–26)	2/4	0/2	1/4	4/4	–	–
		+ Adjuvant	7/13	9.66 (2.54–19.4)	5/7	2/5	5/7	4/7	2/7	1/7
		+ Salvage^	2/13	19 (14–24)	2/2	1/2	0/2	1/2	–	1/2
		^Mean time to BCR (mo): 8 (6–10)								
	8 ( *n* = 8)	Surgery alone	2/8	14.15 (10.3–18)	1/2	1/1	1/2	2/2	–	–
		+ Adjuvant	5/8	6.738 (3.9–12.2)	5/5	3/5	2/5	3/5	1/5	1/5
		+ Salvage^	1/8	30	1/1	0/1	0/1	–	–	1/1
		^Mean time to BCR (mo): 19								
	9 ( *n* = 9)	Surgery alone	2/9	8.2 (6–10.4)	2/2	0/2	0/2	2/2	–	–
		+ Adjuvant	6/9	16.18 (5.5–28)	6/6	3/6	4/6	4/6	–	2/6
		+ Salvage^	1/9	13	1/1	0/1	1/1	–	1/1	–
		^Mean time to BCR (mo): 19								

a Radiation therapy ongoing, or no evidence disease post radiotherapy but completing prolonged course of androgen deprivation therapy.

## Discussion

To date, there has been a paucity of data pertaining to outcomes of radical prostatectomy in Ireland. The only other recently published paper relating to overall outcomes of Irish prostatectomy assesses results of 125 consecutive robot assisted cases with excellent early outcomes at 1 year. 10 Open RRP, however, remains a common operation outside of robotic centers in North America and Europe, and debate continues as to whether an open or robotic approach to radical prostatectomy confers a clear benefit in oncological or functional outcomes. 11 As such, we feel continued publication of open RRP results is both relevant and necessary, and hope that our experience contributes to the gap in the Irish outcomes literature.

Mean PSA at diagnosis for all groups was 9.46 ng/mL (range: 1.06–76.7 ng/mL). This is largely congruent with the mean PSA at diagnosis reported by other authors (mean: 5.4 ng/mL [range: 0.2–66 ng/mL], 12 median 5.6 ng/mL, 13 mean 11.6 ng/mL [standard deviation (SD) 12.4] 14 ) although we note the width of our reported range. While we did not analyze data prior to the local establishment of Rapid Access Prostate Cancer Clinics, it has been previously recognized that these have led to prostate cancer diagnosis at lower mean PSA readings in the Irish population. 4

**Fig. 1:**
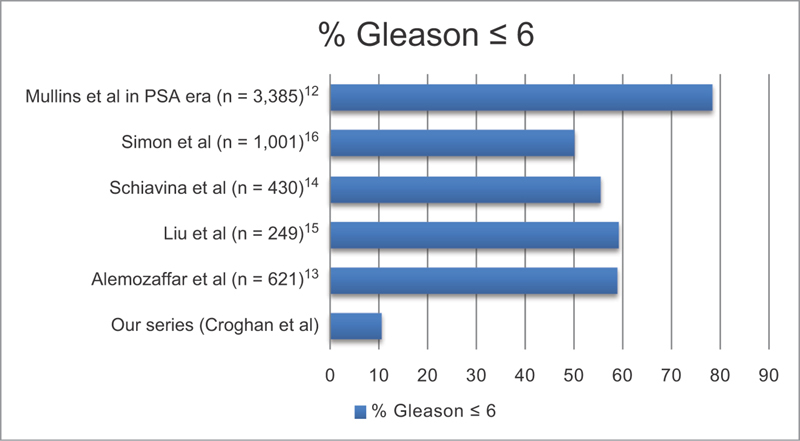
Proportion of patients in radical prostatectomy series with Gleason's score ≤6 disease

With regard to preoperative cancer grading, based on diagnostic trans-rectal ultrasound (TRUS) biopsy, the majority of our RRP patients (139/265, 52.4%) were categorized as harboring Gleason's score 7 (3 + 4 or 4 + 3) disease, followed by Gleason's score 8 (27.38%, *n* = 72). Only a minority (28/265, 10.56%) carried a preoperative diagnosis of Gleason's score 6 disease. We note a different pattern in the literature, with a predominance of patients with preoperative Gleason's scores ≤6 in cohorts undergoing RRP, as outlined in Fig. 1 . 12 13 14 15 16

This may reflect higher grade disease at presentation, an aggressive surgical approach where all high risk patients with nonmetastatic disease are offered surgery and/or the fact that a large proportion of patients with Gleason's 6 disease at our institutions meet the criteria for active surveillance (AS) protocols, and it is the senior author's practice to offer AS to such patients, reflecting a shift in management strategy from that employed in historical series.

Discordance between Gleason score based on diagnostic TRUS biopsy and that reported from the prostatectomy specimen is a recognized phenomenon. Predictors described include the number of biopsy cores and prostate weight, 17 the PSA density 18 and the reporting of the specimen by a uropathologist. 19 The international literature suggests an overall upgrading rate of 29 to 50.5% and an overall downgrading rate of 8.3 to 40%. 17 18 19 20 21 22 Of 263 patients with available pre- and postoperative detailed histopathology available, we found an overall Gleason's upgrade rate of 16.35% (43/263) which is lower than that quoted. This may be reassuring for patients on AS protocols, although we acknowledge the numbers of Gleason's score 6 disease are low in this surgical cohort. Of patients upgraded in our cohort, the largest single group were those with Gleason's 7 (3 + 4) carcinoma on diagnostic TRUS biopsy (17/43, 39.5%). This differs from recent findings by Athanazio et al in a large Canadian study ( *n* = 2,529) where Gleason's 6 to 7 (3 + 4) was the most common alteration in final histology. 20 We noted a 27% (71/263) downgrade rate which is within the range reported. While our specimens are reported as “overall Gleason's score (components),” in acknowledgement of the Gleason's grades incorporated at the 2014 International Society of Urological Pathology (ISUP) consensus conference, 23 we considered Gleason's 7 (3 + 4) and Gleason's 7 (4 + 3) as different grade groups and documented changes between these categories as upgraded or downgraded accordingly.

**Fig. 2:**
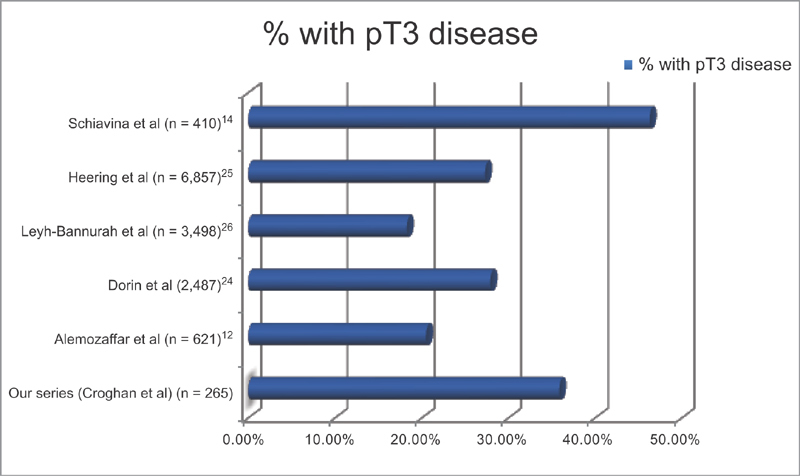
Proportion of patients in radical prostatectomy series with pT3 disease

On histopathological review of the prostatectomy specimen, 170 patients (64.15%) had pT2 disease and 95 patients (35.85%) had pT3 disease. A higher proportion of our patients had pT3 disease than those reported in several large U.S. 13 24 and European 25 26 studies analyzing open RRP data, although lower than those recorded in one Italian study 14 ( Fig. 2 ). This trend toward more locally advanced disease may be related to higher proportions of intermediate and high-risk disease as determined on biopsy Gleason's scoring seen in our patient population.

Much debate surrounds the topic of lymphadenectomy at surgery for prostate cancer. While new approaches, such as sentinel lymph node biopsy may alter the future horizon, 27 pelvic lymph node dissection (PLND) remains the current gold standard approach to nodal staging, with pN1 status an independent predictor of disease recurrence in prostate cancer. 28 29 We performed PLND guided largely by a preoperative MSKCC nomogram predicted rate of lymph node involvement of > 5% as is still the threshold recommended by EAU (European Association of Urology) guidelines. 30 We ultimately had an overall rate of PLND of 44.25% (118/265). Reports of overall rates of PLND performed in RRP populations vary greatly in the literature (26.34–100%) 12 13 31 likely a result of differing proportions of high-risk disease, local policies, and the existence of a variety of nomograms and guidance sources, with the National Comprehensive Cancer Network (NCCN) guidelines 32 and d'Amico risk stratification, 33 for example, also frequently used. Of patients undergoing pelvic lymph node dissection, our 11.86% (14/118) detection rate of nodal metastases is higher than that reported in other studies (reported rates range: 3.4–9.65%) 12 13 14 31 34 This may reflect both higher proportions of more aggressive or locally advanced disease in this cohort compared with that of other series, as discussed above, and also a lower overall PLND rate suggesting a more stringent criteria for proceeding to nodal dissection. We noted no complications specific to lymph node dissection. During the study period, routine protocol was to perform a standard bilateral nodal dissection, with extended dissections (ePLND) performed in selected cases. We are now moving toward extended pelvic lymph node dissection (ePLND) as the standard of care.

Eighteen patients whose final histology was N0 or Nx with a clear surgical margin had a biochemical recurrence. Of this subgroup 11/18 (61.12%) are disease-free postsalvage therapy, 2/18 (11.12%) alive with disease and 5/18 (27.78%) continue to undergo treatment. Mean preoperative PSA was 12.22 (range: 4–30) which was higher than the mean PSA for all patients (9.46). Of the18 patients, 9 (50%) had high-risk Gleason's 8 or 9 disease, and the remainder Gleason's 7 disease. Again, 8/18 (44.45%) had pT3 disease (T3a in 5/8 and T3b in 3/8), all of whom underwent PLND and were staged pN0. Furthermore, 10/18 had pT2 disease (pT2c in 9/10), 5/10 underwent PLND and were staged N0, and 5/10 did not undergo PLND. It is unclear why this cohort experienced biochemical recurrence (BCR), although one possible explanation is that patients harbored low volume nodal metastases in nodes that were not retrieved at PLND. The number of nodes retrieved in the 13 patients in this cohort who underwent PLND (mean: 6.7 [2–26]; median 4 nodes) was similar to that in our overall study population but we acknowledge higher reported nodal retrieval in some studies. 34 We plan to re-evaluate rates of recurrence in pN0R0 patients having implemented ePLND to explore this hypothesis.

PSMs have been shown to be independently associated with increased rates of biochemical recurrence, 35 36 37 38 although their influence on prostate cancer specific mortality is debated. 39 Our overall rate of margin positivity was 26.79% (71/265). Overall rates reported in the literature vary greatly (6.7–45.5%), 12 13 40 41 42 presumably in part due to differing rates of extra capsular extension between studied cohorts. Assessing surgical margins relative to pathological T-stage, we found a PSM rate of 15.2% (26/170) in T2 disease, and 47.37% (45/95) in T3 disease. This compares to literature reported rates of margin positivity in 6.8–34.24% of patients with pT2 disease, and in 23–71.8% of patients with pT3 disease. 14 35 37 41

Of patients with PSMs, 33.8% (24/71) received adjuvant therapy following MDT discussion. In the later years of the study period, adjuvant therapy was administered if further high-risk features in addition to a PSM were present. Of those managed by close clinical and biochemical follow-up as opposed to adjuvant therapy, 14/47 (29.8%) had biochemical recurrence and were referred for salvage therapy and 33/47 (70.21%) were disease-free with no additional treatment at last follow-up. While follow-up is not yet adequate to draw definitive conclusions, we feel that PSMs alone should not prompt automatic administration of adjuvant therapy, and evidence from other experiences would support this. 43

Our results found 243 patients to have completed treatment and have available follow-up data. Of these, 238 (97.9%) were disease-free at last follow-up. Overall, 238/265 (89.81%) of patients were disease-free at the last follow-up (mean 32.24 months), of whom 24/238 (10.08%) had received adjuvant and 17/238 (7.14%) received salvage radiotherapy. Adjuvant/salvage treatment was ongoing in 19/265 (7.17%) of patients, two patients died of unrelated causes, one patient was lost to follow-up and five patients were alive with incurable disease. No patients in this series had died from prostate cancer, although we acknowledge follow-up is short for this outcome. Heterogeneity of populations and disease, variable definitions of biochemical recurrence, and differing practices in administration of adjuvant and salvage radiotherapy make outcomes difficult to compare between studies. Nonetheless, our results to date seem broadly similar to those published in the literature. Early outcomes of anatomical open RRP reported by Catalona et al ( *n* = 925) suggest an overall 5-year progression-free survival probability of 78%. 44 More recent outcomes reported post RRP include those of Alemozaffar et al (3-year recurrence-free survival of 89.9%, *n* = 493), 13 Dorin et al ( *n* = 2487, biochemical recurrence in 11% and clinical recurrence in 3.7% at median follow-up 7.2 years), 24 and Mullins et al ( *n* = 4,478, biochemical recurrence in 749 [16.9%] and local recurrence in 123 [2.8%] at mean 10 years of follow-up [range: 1–29]). Irish outcomes with RALP ( *n* = 125) have shown a biochemical free survival of 92% at 1 year follow-up. 10 We defined BCR as the finding of two PSA readings ≥0.05 ng/mL and demonstrating a rising trend. This is a lower threshold than used by other authors who stipulate, for example, levels of ≥0.2 ng/mL 12 35 43 or higher 37 before declaring BCR. We would typically refer patients fitting our definition for early salvage radiotherapy and therefore have defined BCR as such.

We acknowledge several limitations to our study. Our follow-up data are insufficient to capture cases of late biochemical recurrence, as is a recognized phenomenon. 45 46 For this reason, we keep our patients on annual PSA surveillance as a lifelong measure once they are disease-free for 5 years. Our posttreatment follow-up is further limited in the salvage radiotherapy subgroup due the latency to biochemical recurrence and further time to treatment completion within the study period. We did not record blood loss, transfusion rates, or operative time as these were not uniformly recorded in the computerized records across the hospital sites. Functional outcomes were not assessed. We recognize this as an inherent study weakness; however, these were not recorded in a standardized fashion during the study period and we feel retrospective evaluation of them would therefore lack accuracy. We therefore focused on our primary outcome of oncological results.

## Conclusion

Good oncological outcomes of RRP are seen in this 5-year review with the majority of patients experiencing biochemical-free survival at most recent follow-up. This study adds Irish data to the international literature on prostate cancer disease characteristics in a selected surgical cohort and on outcomes of open radical prostatectomy.
